# Complex Formation Between Ca(II), Mg(II), Al(III) Ions and Salicylglycine

**DOI:** 10.1155/S1565363303000256

**Published:** 2003

**Authors:** Melinda Kilyén, lmre Labádi, Etelka Tombácz, Tamás Kiss

**Affiliations:** 1 Bioinorganic Chemistry Research Group of the Hungarian Academy of Sciences University of Szeged P.O.Box 440 Szeged H-6701 Hungary; 2 Department of Inorganic and Analytical Chemistry University of Szeged P.O.Box 440 Szeged H-6701 Hungary; 3 Department of Colloid Chemistry University of Szeged Hungary

## Abstract

For modelling the interactions of proteins/peptides with hard metal ions the complex formation of salicylglycine (SalGly) with Ca(II), Mg(ll) and AI(III) ions was studied in aqueous solution using pHpotentiometric and UV-vis spectroscopic techniques. Al(lll) ion was found to form more stable complexes with SalGiy than Ca(ll) or Mg(ll) ions. While AI(III) ion forms various 1:1 complexes of different protonation states in the pH range 2-7, Ca(ll), Mg(ll) ions seem to interact with SalGly only in the basic pH range and form mixed hydroxo species MLH_-1_ at pH ~ 8. According to the UV-vis spectroscopic measurements in the species MLH_-1_ the carboxylate-O^-^ atom and the phenolate-O^-^ coordinate to the metal ions. SaIGiy is able to keep Al(lll) in solution through inner and outer sphere coordination to metastable amorphous AI(OH)_3_ particles. Deprotonation of the peptide amide Nil does not occur in these systems.


### Complex Formation Between Ca(II), Mg(II), Al(III) Ions and Salicylglycine

Melinda Kilyén[a], Imre Labádi[b], Etelka Tombácz[c] and Tamás Kiss[a,b]*

[a] *Bioinorganic Chemistry Research Group of the Hungarian Academy of Sciences, University of Szeged, P.O.Box 440, H-6701 Szeged, Hungary, Fax: 36-62-420505, e-mail:tkiss@chem.u-szeged.hu,*

[b] *Department of Inorganic and Analytical Chemistry, University of Szeged, P.O.Box 440, H-6701 Szeged, Hungary*

[c] *Department of Colloid Chemistry, University of Szeged, Hungary*

### ABSTRACT

For modelling the interactions of proteins/peptides with hard metal ions the complex formation of salicylglycine (SalGly) with Ca(II), Mg(II) and Al(III) ions was studied in aqueous solution using pH-potentiometric and UV-vis spectroscopic techniques. Al(III) ion was found to form more stable complexes with SalGly than Ca(II) or Mg(II) ions. While Al(III) ion forms various 1:1 complexes of different protonation states in the pH range 2–7, Ca(II), Mg(II) ions seem to interact with SalGly only in the basic pH range and form mixed hydroxo species $MLH_{-1}$ at pH ~ 8. According to the UV-vis spectroscopic measurements in the species $MLH_{-1}$ the carboxylate-$O^-$ atom and the phenolate-$O^-$ coordinate to the metal ions. SalGly is able to keep Al(III) in solution through inner and outer sphere coordination to metastable amorphous $Al(OH)_3$ particles. Deprotonation of the peptide amide NH does not occur in these systems.

**Keywords:** Salicylglycine, Ca(II) complexes, Mg(II) complexes, Al(III) complexes, Speciation

### INTRODUCTION

The active site of a metalloenzyme usually consists of several donor atoms or groups in a special arrangement to be able to bind metal ions. The metal centre formed is responsible for the activity of the enzyme. Redox or non-redox metal ions can be involved in metalloenzymes depending on the type of reactions they catalyse. In order to understand the role of the metal ions in mechanism of enzymes and developing new synthetic metalloenzymes, it is necessary to know more about the interaction of metal ions with peptides and proteins. Large number of metalloenzymes have been crystallised and their structure characterised by X-ray diffraction. Often the dynamic structures and catalytic properties of the enzyme in solution are clarified by multinuclear NMR techniques, when changes in the active site of the enzyme during the catalytic cycle are monitored. Besides this approach, the structural and functional modelling of the active site of metalloenzymes is another way to obtain the desired information. Zn(II), Fe(II), Mn(II), Cu(II), Ca(II) and Mg(II) metal ions are frequently present in metalloenzymes, hence their complexes with relevant model molecules are the most studied. There are also many other metal ions, which are not essential elements, but they may influence the activity of enzymes. One of these metal ions is Al(III). The detrimental role of Al(III) in many biological, enzymatic processes is well documented /1-4/. In many cases the toxicity of Al(III) is linked to its ability to replace Ca(II) or Mg(II) ions in their biological environment and in this way to interfere with their reactions in biological systems /5/. In this paper the coordination behaviour of Salicylglycine (SalGly) with Ca(II), Mg(II) and Al(III) has been investigated by pH-metry and UV-vis spectroscopic method.

SalGly (Figure 1) is a hydrolysis product resulting from glycine conjugation with salicylic acid and it is a metabolite of the widely used analgesic known as aspirin /6/.

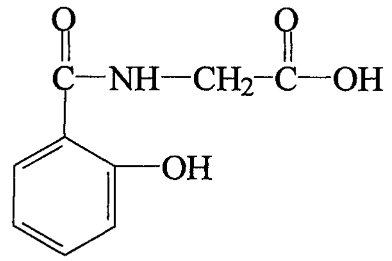

**Fig. 1:** Formula of the ligand SalGly

SalGly as a dipeptide analogue may be a good model compound to study the metal-peptide/protein interaction, which can have further applications in clarifying the interactions of the toxic Al(III) in biological systems. SalGly contains a carboxyl group, a peptide amide group and a phenolic OH group as potential donors for the metal ion binding. The phenolic OH group may be a suitable anchoring donor for hard metal ion and may play a crucial role in the deprotonation and subsequent coordination of the peptide amide. Stability constants of SalGly with Cu(II), Ni(II), Zn(II), Co(II) /7-9/ and VO(IV) ions /10/ were determined previously. SalGly proved to be a relatively strong binder of these transition metal ions. With Cu(II) and VO(IV) ions an $MLH_{-1}$ complex is predominantly formed in the pH range 4–6. In this complex the ligand coordinates to the metal ion trough the phenolate, carboxylate oxygen atom and the deprotonated peptide nitrogen through a (5+6)-membered joint chelate system. With Ni(II) and Zn(II) ions SalGly forms mononuclear complexes ML and $MLH_{-1}$. In the complex $MLH_{-1}$, SalGly binds to these metal ions in a bidentate way through the phenolate and carboxylate donors and a proton is liberated from a coordinated water molecule.

These studies reveal that Cu(II) and VO(IV) ions favour deprotonation and coordination of the peptide amide, while it does not occur in the Ni(II) and Zn(II) complexes.

### EXPERIMENTAL SECTION

### Reagents:

Salicylglycine (2-hydroxyhippuric acid), of highest analytical purity (Aldrich product) was used without further purification. The exact concentration of the ligand solution was determined by potentiometric titration using the Gran method /11/. The Ca(II) and Mg(II) solutions were prepared from Fluka products, $CaCl_2 \cdot 2H_2O$, and $MgCl_2 \cdot 6H_2O$, puriss. >99% quality and their concentrations were checked by complexometric titrations. The Al(III) stock solution was prepared from recrystallized $AlCl_3 \cdot 6H_2O$ and its metal concentration was determined gravimetrically via its oxinate. The stock solution contained 0.1 M HCl to prevent hydrolysis of Al(III).

### Potentiometric Measurements:

The stability constants of the proton and metal ion complexes of the ligand were determined by pH-potentiometric titrations of 10 mL samples. The ligand concentration was 0.002 M or 0.004 M. Titrations were performed with a 0.2 M carbonate-free KOH solution of known concentration under a purified argon atmosphere. The measurements were carried out at metal ion to ligand ratios of 0:1, 0:4, 1:1, 1:2, 1:4 for Ca(II) and Mg(II) ion, while at 0:1, 0:4, 1:1, 1:2, 1:4, 1:8, 1:11, 1:16 for Al(III) ion. The ionic strength of all solutions was adjusted to 0.2 M with KCl and the temperature was 25 ± 0.1°C. The titrations were performed until precipitation occurred in the systems. The reproductibility of the titration curves was within 0.01 pH units through the whole pH range. When equilibrium could not be reached in 10 min, titration points were omitted from the calculations. The pH range studied was 2–11 for Ca(II), Mg(II) ion and until precipitation occurred for Al(III) ion. The pH was measured with a Molspin Automatic Titrator equipped with Metrohm 6.0234.100 type combined glass electrode, which was calibrated for the hydrogen ion concentration according to Irving at al. /12/. The stability constants ($\beta_{pqr} = [M_pL_qH_r]/[M]^p[L]^q[H]^r$) were calculated with the aid of the PSEQUAD computer program /13/. The fitting parameter, which characterises the goodness of the fit and represents the average difference between experimental and calculated titration curves, is expressed in mL of the titrant. The stability constants used for the hydroxo species of Al(III), Mg(II) and Ca(II) were taken from ref. /14,15/ and corrected to I = 0.2 using the Davies equation: –5.49 for $[AlH_{-1}]^{2+}$,–10.91 for $[AlH_{-2}]^+$, –13.54 for $[Al_3H_{-4}]^{5+}$, –108.62 for $[Al_{13}H_{-32}]^{7+}$, –23.40 for $[AlH_{-4}]^-$, and –11.91 for $[MgH_{-1}]^+$, –37.09 for $[Mg_4H_{-4}]^{4+}$ and –14.14 for $[CaH_{-1}]^+$.

### Spectrophotometric measurements:

UV-vis spectra were recorded on a HP 8452A diode array spectrometer in 0.5 cm quartz cell in the 200–500 nm spectral range on solutions containing $2 \cdot 10^{-4}$ M ligand, of metal ion to ligand molar ratios of 0:1, 1:2, 1:8. The pH range studied was 3-12. The ionic strength was adjusted to 0.2 M with KCl.

### Dynamic light scattering measurements:

Dynamic light scattering (DLS) measurements were performed using a ZetaSizer 4 (Malvern, U.K.) apparatus operating at $\lambda = 633$ nm produced by a He-Ne laser at angle 90° at 25 ± 0.1 °C. The light scattering was measured in 10 mL samples containing Al(III) alone and Al(III) and the ligand at the same concentration as for potentiometric measurements ($c_{ligand} = 4 \cdot 10^{-3}$ M) and at 1:8 the metal ion to ligand ratio. The pH of dilute systems was adjusted in the pH range 4–7 and measured directly before the sample was placed into the quartz cell. The pH-dependent particle aggregation was measured at 0.2 M KCl constant ionic strength.

### RESULTS AND DISCUSSION

### Potentiometric Measurements

Potentiometric titrations of SalGly indicate the stepwise dissociation of two protons in the measurable pH range, one from the carboxylic group with pK = 3.38 and one from phenolic hydroxyl group with pK = 8.11. The measured values are in reasonably good agreement with the earlier literature data (see Table 1).

**Table 1**

Acid dissociation constants (pK, 3SD values are given in parentheses) of SalGly ligand at 25 °C

| pK(COOH) | pK(OH) | Conditions | Literature |
|---|---|---|---|
| 3.44 | 8.24 | 0.2 M KCl | /7/ |
| 3.34 | 7.91 | 0.1 M $NaNO_3$ | /8/ |
| 3.37 | 8.16 | 0.2 M KCl | /10/ |
| 3.38(4) | 8.11(2) | 0.2 M KCl | Present work |

A comparison of the pK values of SalGly with those of glycine, pK(COOH) = 2.37 and pK($NH_3^+$) = 9.60 and glycyl-glycine with pK(COOH) = 3.21 and pK($NH_3^+$) = 8.13 shows that the pK = 3.38 value of carboxylic group of SalGly is close to that of the dipeptides /16/. The pK = 8.11 value of phenolic-OH group of SalGly is close to that of the terminal amino group of GlyGly. Accordingly, concerning the donor group arrangement and the basicity of the donors, SalGly is a good model for peptides, however, the neutral $-NH_2$ terminus is replaced by a negatively charged $O^-$ donor, which may be a more suitable anchor for hard metal ions.

The stability constants (log(β) calculated by the joint evaluation of the titration curves obtained at various metal ion to ligand ratios for Ca(II)–, Mg(II)– and Al(III)–SalGly systems are listed in Table 2.

**Table 2**

Proton and stability constants (log β, 3SD values are given in parentheses) of Ca(II), Mg(II) and Al(III) complexes with SalGly at I = 0.20 M (KCl) and 25°C

| log β | Ca(II) | Mg(II) | Al(III) |
|---|---|---|---|
| MLH | | | 10.78(6) |
| ML | | | 7.65(2) |
| $MLH_{-1}$ | –8.42(9) | –9.19(6) | 2.75(4) |
| $MLH_{-2}$ | | | –2.18(5) |
| Fitting [a] | 0.0047 | 0.0053 | 0.0096 |
| No. of points | 302 | 370 | 623 |

[a] Average difference between experimental and calculated titration curves expressed in mL of the titrant.

Evaluation of the titration data for Ca(II)–, Mg(II)–SalGly led to a model including only a single mononuclear hydroxo complex $MLH_{-1}$ (or more precisely ML(OH)), which occurs at pH > 8. Other mononuclear species MLH, ML were rejected by the computer program. Due to the high proton competition on the phenolate site, coordination occurs only at low hydrogen ion concentration in overlapping processes of the hydrolysis of the metal ion resulting in the formation of a ternary mixed hydroxo complex. The interaction of SalGly with these metal ions is rather weak (their stability constants are similar), represented by the fact that the extent of complexation hardly reaches 10 % at a ten fold excess of ligand at pH ~ 8. pH-potentiometry is not the best method to determine such low stability constants, and thus very few stability data have been published in the literature on alkali-metal complexes /17-20/. The clearly observed higher absorbance in the UV spectra of the ligand in the presence of metal ions, as compared with that in the absence of Mg(II) or Ca(II) ions, unambiguously prove the coordination of the metal ions.

The complex formation with Al(III) is more complicated because of the more complex hydrolytic equilibrium of the Al(III) ion. In order to prevent precipitation of the neutral $Al(OH)_3$, pH-metric measurements were performed at high excesses of ligand, too. Depending on the metal ion to ligand ratio, precipitation occurred at different pH values at pH ~ 4.7 for 1:1 metal to ligand ratio, in the range of pH 5.2–5.5 for 1:2 and 1:4 metal to ligand ratio, while only at pH ~ 7 for the 1:8 or higher metal ion to ligand ratio. The equilibrium titration data for Al(III)–SalGly system were evaluated by a speciation model including mononuclear complexes in different protonation states AlLH, AlL, $AlLH_{-1}$ and $AlLH_{-2}$ (see Table 2). The formation of various dinuclear species was also tested in the evaluation, but these species were always rejected in the calculation procedure. In the pH ranges indicated above, complex formation was fast; pH equilibrium was reached in less than 5 min, thus formation of oligonuclear complexes in such low Al(III) concentrations seemed to be negligible. The interaction of Al(III) with SalGly is significantly stronger than with Ca(II) and Mg(II) ions. As seen in the speciation curves (Figure 2) complex formation is practically complete by pH 5 at an eight-fold excess of ligand.

Figure 2 reveals that complex formation starts at pH ~ 2 with a protonated complex AlLH. In this

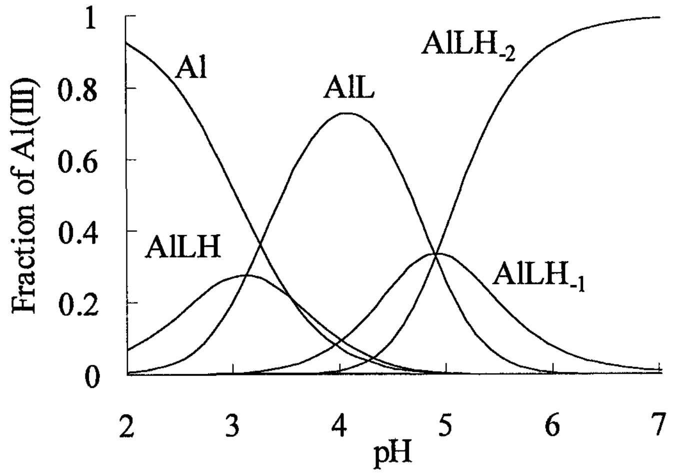

**Fig. 2:** Species distribution curves of the Al(III)–SalGly 1:8 system as a function of pH, $c_{SalGly}$ = 0.004 M

complex the ligand probably coordinates in a monodentate way through the terminal $COO^-$ function (see structure I, Chart 1), with the possible chelation through the peptide carbonyl (see structure II, Chart 1) as was suggested for the corresponding Cu(II) complex too /7/.

Increasing the pH, the protonated species AlLH undergoes stepwise deprotonations with a pK = 3.13, 4.90 and 4.93 and forms finally the complex $AlLH_{-2}$. The liberation of these protons occurs in overlapping processes from the phenolic-OH group and the coordinated water molecules, resulting in the formation of different binding isomers shown in Chart 1. For example liberation of the first proton may occur (i) from the phenolic-OH group, resulting in the bidentate ($COO^-$, $O^-$) coordination of the ligand (See Structure III in Chart 1) with a possible involvement of the peptide carbonyl through the formation of a 6+6 membered joint chelate system /7/ (see Structure IV in Chart 1), or (ii) from one of the coordinated water molecules, which assume monodentate carboxylate coordination of the ligand, with protonated phenolic-OH group with a possible involvement of the peptide carbonyl group. In these latter cases the low pK(AlL) value of 3.13 may be interpreted by the formation of a strong hydrogen-bonding between the phenolic-OH and the coordinated $OH^-$ (see Structure V in Chart 1) and/or a change in the coordination geometry from octahedral to tetrahedral /21, 22/. Liberation of the next proton will result in the formation of a mixed hydroxo species with either bidentate ($COO^-$, $O^-$) or tridentate ($COO^-$, CO, $O^-$) coordination of the ligand (Structures VI and VII, respectively in Chart 1).

The rather low pK value of species $AlLH_{-1}$ (pK($AlLH_{-1}$) = 4.90), which is 0.59 log unit lower than the pK of the $[Al(H_2O)_6]^{3+}$ = 5.49 may suggest also the presence of a hydrogen bonding with the coordinated $OH^-$. On increasing the pH a further deprotonation takes place with (pK($AlLH_{-2}$) = 4.93). Probably, a second coordinated water molecule dissociates and the mixed bis hydroxo complex $AlL(OH)_2$ is formed. Another alternative interpretation of this deprotonation step is the assumption of an outer-sphere complex formation between the metastable non-precipitated $Al(OH)_3$ and the protonated (on the phenolic function) form of the ligand $HL^-$. Al(III)–ligand systems frequently exist in metastable states when solubility product should predict precipitation; the solution may be clear even for days /23/. At pH ~ 6 the slight precipitation observed

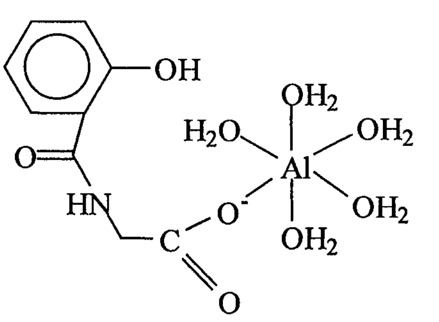

I

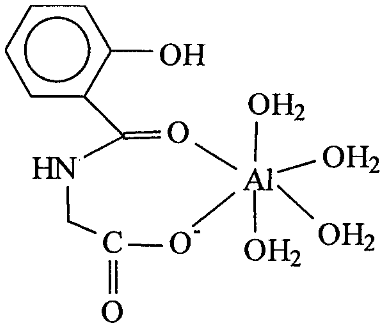

II

AlLH

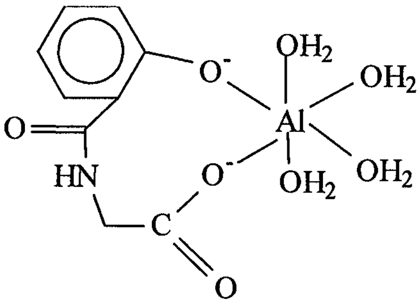

III

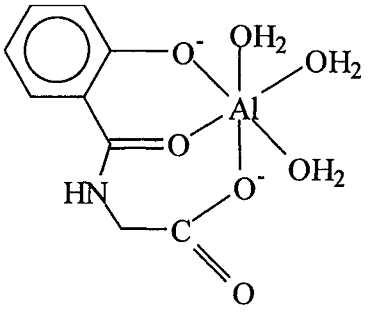

IV

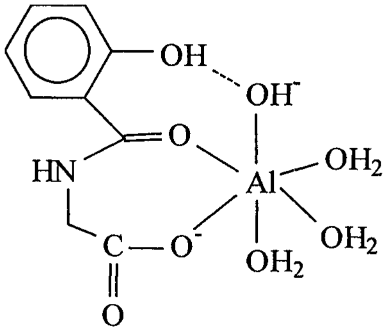

V

AlL

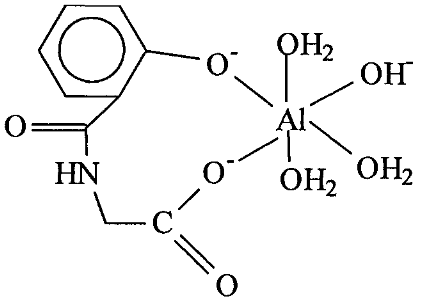

VI

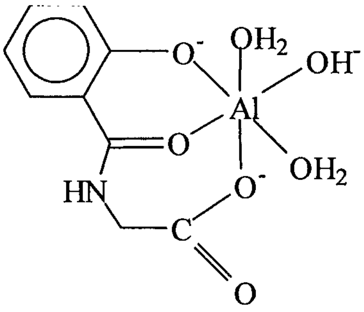

VII

$AlLH_{-1}$

**Chart 1**

at low excess of ligand was completely redissolved by pH ~8, resulting again in clear solution. The stability constants of the Al(III) species are higher than those of the analogous Cu(II), Ni(II) and Zn(II) complexes of the SalGly /7/. This can be explained by the higher charge of the $Al^{3+}$, which results in a significantly higher electrostatic contribution to the stability of Al(III) complexes and points to the primarily electrostatic character of the interaction.

### Spectrophotometric measurements

The suggested binding modes of the different complexes were confirmed by spectrophotometric measurements. Using this method the protonation state of the phenolic-OH group could be monitored by UV-vis spectrometry as the phenolic-OH group has a characteristic band at 298 nm, which is shifted to 326 nm upon deprotonation. The metal binding strength of the phenolate group is considerably higher than in the protonated form /24, 25/. The UV-vis spectra of SalGly in the absence and in the presence of Al(III) at different pH values are depicted in Figure 3 (a and b).

As seen in Fig. 3a, the phenolic OH is protonated in acidic solution and gives a band between 270 and 340 nm with maximum at 298 nm in the pH range 3–6. On increasing the pH, a new band develops at 326 nm, corresponding to the deprotonation of phenolic OH group. The isobestic point observed at 307 nm indicates two species in equilibrium: the phenolic function being either in protonated or deprotonated form.

The presence of Ca(II) or Mg(II) ion has only a slight effect on the UV-vis spectra of the ligand, indicating that these metal ions can induce deprotonation of the phenolic OH only weakly. At pH > 6 when the ligand starts to deprotonate by itself, these metals are able to bind the ligand in the $MLH_{-1}$ complex by

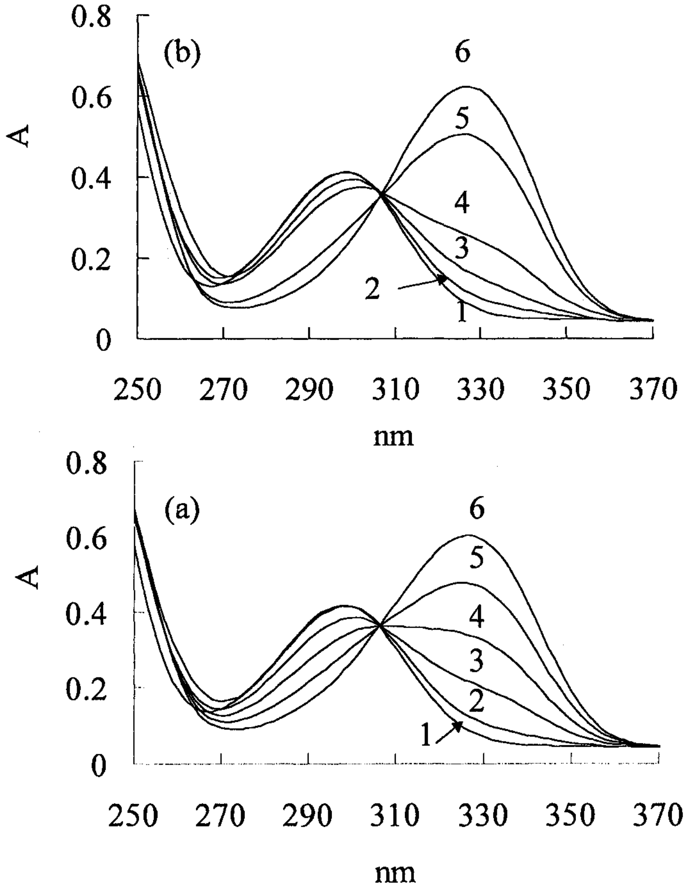

**Fig. 3:** The UV-vis spectra at different pH values: (a) the ligand SalGly alone at (1) pH < 5; (2) pH ~ 6; (3) pH ~ 8; (4) pH > 8.5; (5) pH ~ 9; (6) pH ~ 10; and (b) the Al(III)–SalGly system at: (1) pH ~ 3; (2) pH ~ 4, 6, 7; (3) pH ~ 5; (4) pH ~ 8; (5) pH ~ 9; (6) pH ~10; $c_{SalGly}$ =0.002 M and $c_{Al(III)}$=0.00025 M.

chelation through the carboxylate and phenolate groups.

The UV-vis spectra of Al(III)–SalGly system recorded at different values in the pH range 3–12 also consists of two bands at 298 nm and 326 nm (see Figure 3b). In acidic solution (pH < 4) the band of phenolic OH group (298 nm) is the dominant one. The absorbance of the band at 326 nm, characteristic to the phenolate group, increases upon increasing the pH up to ~5 (see Figure 3b) and results in the disappearance of the band at 298 nm. This indicates the Al(III) induced deprotonation of the phenolic OH and the subsequent coordination to Al(III). At pH > 5, when the species $AlL(OH)_2$ starts to be formed the absorbance belonging to the phenolate group decreases, which can be explained by the assumption of the re-protonation of the phenolate group accompanied by its release from the coordination sphere of Al(III).

In the pH range 3–6 the formation of complex $AlLH_{-1}$ can be detected by both UV-vis spectroscopy and pH-potentiometry. Comparing the species distribution curves (see Figure 2) with the change of the absorbance measured at 326 nm as a function of pH (see Figure 4), we can conclude that only the species $AlLH_{-1}$ contains deprotonated and Al(III) coordinated phenolate group. Both the potentiometric and the spectrophotometric measurements show a maximum at pH ~ 5, when approximately 30% of Al(III) is complexed in the species $AlLH_{-1}$ (Figure 2). Accordingly, in this complex the bidentate ($COO^-$, $O^-$) coordination of the ligand is the most likely binding mode (see Structures III and IV in Chart 1). Interestingly enough, the direct coordination of the phenolate group of SalGly to Al(III) in species $AlL(OH)_2$ is not confirmed by UV-vis spectroscopy. The re-protonation of the phenolate group in the formation pH range of species $AlL(OH)_2$ can occur only through the displacement of the phenolate group from the coordination sphere of the Al(III) by a further $OH^-$. Accordingly, the binding mode of $AlL(OH)_2$ may be the direct monodentate $COO^-$ coordination of the ligand to the metastable form of $Al(OH)_3$ and solubilization and stabilization of the species through outer-sphere hydrogen bonding with the protonated phenolic-OH and the carbonyl-O functions, resulting in a species written precisely by the formula $Al(OH)_3(HL)$. Since the UV-vis spectra of the SalGly in the presence and the absence of Al(III) does not appreciably differ in the pH range 7–12, where the ligand is completely displaced by $OH^-$ resulting in the formation of the very stable $[Al(OH)_4]^-$,

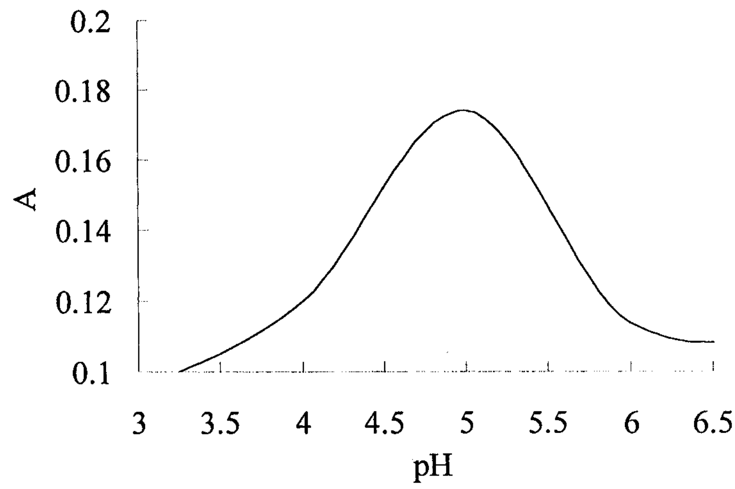

**Fig. 4:** The pH dependence of the absorbance measured at 326 nm in the Al(III)–SalGly 1:2 system, $c_{SalGly}$ = 0.002 M.

deprotonation of amide nitrogen cannot be assumed in the systems studied. This suggests that the phenolic-OH group of SalGly is an efficient donor group to prevent hydrolysis of these metal ions but not strong enough to promote amide deprotonation in the Ca(II)–, Mg(II)–, Al(III)–SalGly systems. Perhaps, more negatively charged O donor groups in suitable arrangement are required, if at all, to promote the deprotonation and participation of the amide-$N^-$ in binding such hard metal ions.

### Dynamic light scattering measurements

In order to study the aggregation processes resulting in precipitation at pH > 6 in the Al(III)–SalGly system, and to clarify more precisely the binding mode in the complex $AlLH_{-2}$, dynamic light scattering measurements were also carried out. The aggregation processes in dilute suspensions can be characterised by particle size determination. Dynamic light scattering method (DLS) can provide reliable particle size data even, when the system is undergoing coagulation /26/. The complete elucidation of the aggregation features of the Al(III)–SalGly system would need extensive DLS measurements, including kinetic studies. However, as the aggregation process is very complicated and the information that these results may provide is only approximate and indirect concerning the Al(III) binding behaviour to the ligand SalGly, we did not attempt to explore this field in depth, but used DLS only to obtain several basic characteristics for the binding mode in $AlLH_{-2}$.

Comparing the results obtained for the samples containing Al(III) alone and Al(III) and SalGly at a 1:8 ratio at different pH values, the formation of solid particles, presumably $Al(OH)_3$ was observed at different pH values: at pH ~ 4.7 for samples containing Al(III) alone, at pH ~ 6 for the Al(III)-ligand 1:8 system. This is the pH range where the complex $AlLH_{-2}$ predominates in solution (see Figure 2). In the absence of SalGly approximately 2-6 times bigger particles were formed than in the Al(III)–SalGly system. The adsorption or outer-sphere binding of the ligand on the surface of $Al(OH)_3$ nanoparticles was pH dependent; the extent of adsorption of SalGly increased with increasing pH. These observations suggest that the direct or outer-sphere coordination of the ligand to Al(III) has a great influence on the aggregation behaviour of the $Al(OH)_3$ nanoparticles. Namely, SalGly through the formation of the proposed outer-sphere type complex $AlLH_{-2}$ or more precisely $Al(OH)_3 \cdot HL$ hinders aggregation.

### CONCLUSION

The speciation studies indicate a weak interaction of SalGly with Ca(II), Mg(II) and Al(III) ions. Mononuclear 1:1 complexes are formed in these systems. In the case of Ca(II) and Mg(II) only the mixed hydroxo species $MLH_{-1}$ (more precisely ML(OH)) occurs at pH > 8, while in the Al(III)–SalGly system various 1:1 complexes of different protonation states are formed in the pH range 2–6.5. In the complexes AlLH and AlL the phenolic-OH group of SalGly remains protonated (see Structures II and IV in Chart I). The UV-vis spectral changes (see Figure 4) provide convincing evidence that the phenolic-OH is deprotonated in the species $AlLH_{-1}$. In this complex SalGly is possibly bound in a tridentate ($COO^-$, CO, $O^-$) way and an $OH^-$ is also bound to the metal ion (see Structure VII in Chart I). In the species $AlLH_{-2}$, formed at pH > 5.5, besides the direct coordination through the $COO^-$ donor the phenolic-OH is assumed to be in hydrogen bonding with the metastable hydrolytic product of Al(III). The question is whether this type of complex formed between a nanosize particle and the ligand $HL^-$ is stochiometric or not. Probably the association is equimolar: $Al(OH)_3 \cdot HL$. Similar outer-sphere interaction was observed in the phosphate uptake by $Al(OH)_3$ precipitated *in situ*, or by aged $Al(OH)_3$ /27/. Depending on the excess of ligand, precipitation occurred in the pH range 4.7–7.5, which was hindered by the presence of SalGly. At pH~8 the precipitate dissolved through the formation of $Al(OH)_4^-$. No indication of deprotonation of the peptide amide group was observed in this pH range.

The results obtained indicate that Al(III) may be kept in solution not only by direct coordination, but also in metastable forms through outher sphere complexation in biologycal systems.

### ACKNOWLEDGEMENTS

This research was supported by the Hungarian Science Research Fund (OTKA No T37385). The authors wish to express thanks to Ms. Gy. Erdősi (University of Szeged) for her help in the experimental work.

### REFERENCES

1. W.R. Harris, G. Berthon, JP. Day, C. Exley, T.P. Flaten, W.F. Forbes, T. Kiss, C. Orvig, P. Zatta, *J. Toxicol. and Environm. Health* **48**, 543 (1996)
2. C. Exley, N.C. Price, J.D. Birchall, *J. Inorg. Biochem.* **54**, 297 (1994)
3. P. Zatta, P. Zambenedetti, V. Bruna, B. Filippi, *NeuroReport* **5**, 1777 (1994)
4. M.J. Strong, M.R. Garutto, J.G. Joshi, W.R. Mundy, T.J. Shafer, *J. Toxicol. Environm. Health* **48**, 599 (1996)
5. C. Exley, *J. Inorg. Biochem* **76**, 133 (1999)
6. G. Gibson and P. Skett, *Introduction to Drug Metabolism*, Chapman and Hall, New York (1986)
7. E.B. Gonzales, N.N. Daeid, K.B. Nolan, E. Farkas, *Polyhedron* **13**,1495 (1994)
8. A. Bavoso, L. Menabue, M. Saladini, M. Sola, *Inorg. Chim. Acta* **244**, 207 (1996)
9. K.B. Nolan, A.A. Soudia, *Inorg. Chim. Acta* **230**, 209 (1995)
10. T. Kiss, K. Petrohán, P. Buglyó, D. Sanna, G. Micera, J. Costa Pessoa, J. Madeira, *Inorg. Chem.* **37**, 6389 (1998)
11. G. Gran, *Acta Chem. Scand.* **4**, 559 (1950)
12. H.M. Irving, M.G. Miles, L.D. Petit, *Anal. Chim. Acta* **38**, 475 (1967)
13. L. Zékány, I. Nagypál, G. Peintler, PSEQUAD for Chemical Equilibria, Technical Software Distributions, Baltimore (1991)
14. L.O. Öhman, S. Sjöberg, *Acta Chem. Scand.* **A36**, 47 (1982)
15. R.M. Smith, A.E. Martell, Critical Stability constants, vol. 4, Plenum, New York (1976)
16. L.D. Pettit, K.J.Powell, Stability constant database, Academic Software-IUPAC, London (1993)

17. D. Midgeley, *Chem. Soc. Rev.* **4**, 549 (1975)
18. P.G. Daniele, A. De Robertis, C. De Stefano, S. Sammarano, *J. Chem. Soc. Dalton Trans.* 2353 (1985)
19. A. De Robertis, C. De Stefano, C. Rigano, S. Sammarano, R. Scarcella, *J. Chem. Res.* **43(S),** 629 (M) (1985)
20. L. Harju, *Talanta* **34**, 817 (1987)
21. R.B. Martin, *Met. Ions Biol. Syst.* **24,** 1 (1988)
22. T. Kiss, I. Sóvágó, R.B. Martin, *Inorg. Chem.* **30,** 2130 (1991)
23. A.E. Martell, R.J. Motekaitis, R.M. Smith, *Polyhedron* **9**, 171 (1990)
24. F. Cariati, L. Erre, G. Micera, A. Panzanelli, G. Ciani, A. Sironi, *Inorg. Chim. Acta* **80**, 57 (1983)
25. G. Micera, L. Strinna Erre, P. Piu, F. Cariati, G. Ciani, A. Sironi, *Inorg. Chim. Acta* **107**, 223 (1985)
26. H. Holthoff, S.U. Egelhaaf, M. Borkovec, P. Schurtenberger, H. Sticher, *Langmuir* **12**, 5541 (1996)
27. L. Lijklema, *Environ. Sci. Techn.* **14**, 537 (1980)

